# Analysis of Bending Deformation and Stress of 6063-T5 Aluminum Alloy Multi-Cavity Tube Filled with Liquid

**DOI:** 10.3390/ma17133230

**Published:** 2024-07-01

**Authors:** Xinlong Zhang, Zhaosong Jiang, Shuang Zhao, Xiaodong Xie, Jiang Xiao, Xueyan Liu, Zhe Wu, Yang Zhang

**Affiliations:** 1 College of Mechanical and Electrical Engineering, Northeast Forestry University, Harbin 150040, China; zhangxinlong2009@nefu.edu.cn (X.Z.); jzs@nefu.edu.cn (Z.J.); zslxy2022@nefu.edu.cn (S.Z.); 2022111925@nefu.edu.cn (X.X.); xiao7777@nefu.edu.cn (J.X.); liuxueyan@nefu.edu.cn (X.L.); wuzhe@nefu.edu.cn (Z.W.); 2 College of Science, Northeast Forestry University, Harbin 150040, China

**Keywords:** aluminum alloy multi-cavity tube, forming by bending, liquid-filled forming, wall thickness, cross-section distortion

## Abstract

The production of aluminum alloy multi-lumen tubes primarily involves hot bending formation, a process where controlling thermal deformation quality is difficult. Specifically, the inner cavity wall of the tube is prone to bending instability defects under the bending stress field. To address these challenges in the bending deformation of aluminum alloy multi-lumen tubes, a multi-lumen liquid-filled bypass forming method is proposed in this paper. This study focuses on the 6063-T5 aluminum alloy double-lumen tube as the research object. The liquid-filled bending deformation behavior of the aluminum alloy double-lumen tube was investigated, and the deformation theory of the aluminum alloy double-lumen tube was studied. Through experimental and numerical simulation methods, the influence of support internal pressure, bending radius, and tube wall thickness on the liquid-filled bending deformation behavior of the double-lumen tube was examined. The results indicate that when the value of internal pressure was 7.5 MPa, the straightening of the outer wall was improved by 2.51%, the thinning rate of wall thickness was minimized, and the internal concave defect was effectively suppressed. The liquid-filled bending method provides a promising new approach for the integrated bending and forming of multi-lumen tubes.

## 1. Introduction

To address the “dual-carbon” strategy and adapt to the trend of environmental protection, lightweighting technology is applied to the aerospace and automotive industries to reduce energy consumption and emissions. Automobiles and airplanes leverage component lightweighting to achieve strength enhancement and mass reduction, which has become a mainstream approach. There are two methods to achieving lightweighting: using high-strength, lightweight materials such as alloys [1,2,3] and composites to replace conventional carbon steel, or employing hollow, variable-section, and variable-thickness complex structures instead of conventional welded and riveted components. Material and structural weight reduction in many areas has significant potential, and there is still much room for progress. High-strength aluminum alloy multi-lumen tubes, as structural support parts, offer improved support and better mechanical properties and material continuity compared to traditional welded fittings and cold-formed parts.

Aluminum alloy multi-lumen tubes [4] offer a unique combination of weight reduction, making them an ideal structural support member for aerospace and automotive industries. Unlike traditional bending tube fittings, which have a single lumen, multi-lumen tubes provide dual characteristics with better support. This is because the lumen in the tube helps to distribute the stress more evenly, reducing the risk of stress concentration and improving the overall mechanical properties of the tube. Additionally, the use of multi-lumen tubes can improve material continuity, as the tube is made from a single piece of material, eliminating the need for welding or cold-forming. This results in a more consistent and reliable product with better performance and service life [5,6].

The traditional tube bending process involves several methods, including drawing forming, bending forming, roll forming, and push bending forming. While these methods offer high processing efficiency for plates, bars, and tubes, they can also introduce residual stresses, wrinkles, cracking, cross-sectional distortion, and uneven plastic deformation, among other molding defects. To address these issues, CNC processing technology has been widely adopted to improve the quality of tube bending. This approach allows for the formation of large-diameter and thickness ratios of thin-walled tubes, reducing the occurrence of wrinkles and cracks. However, the CNC bending process is complex and its difficulty varies greatly depending on factors such as wall thickness, tube diameter, material, and relative bending radius.

Hydroforming is a versatile metal-forming process that offers several advantages over conventional forming methods [7,8,9]. Its ability to produce complex geometries with high precision and accuracy makes it essential in various industries [10,11]. The liquid-filled bending method, an extension of hydroforming, was developed to address challenges associated with traditional bending processes [12]. This method controls the support pressure applied to the formed fittings by adjusting the liquid pressure inside the tube. This precise control reduces defects such as cross-sectional distortion, folding, and cracking during bending, thereby expanding the bending and forming limits of the tube fittings [13,14]. The unique advantages of liquid-filled forming technology have made it a leading choice for tube fitting plastic bending. It has been widely adopted in the manufacturing of tube fittings [15,16].

Cui et al. [17] proposed a method for regulating the elastic deformation of the mold and improving the dimensional accuracy of rectangular tubular parts during hydroforming. This method involves controlling the forming pressure to achieve precise dimensional control. Experiments conducted on 6063 aluminum alloy tubes demonstrated the successful implementation of this method, enabling the production of rectangular tubular parts with controlled cross-sectional dimensions. By adjusting the forming pressure, the elastic deformation of the mold can be managed, compensating for the rebound of the tube. This approach significantly enhances the flexibility of the hydroforming process and leads to improved dimensional accuracy for tubular components with irregular cross-sections. Song et al. [18] investigated the use of the hydroforming method to pressurize tubes, which could reduce the axial pressure required during tube forming and improve the stability against bending deformation and wrinkling in tube fittings. Xie et al. [19] introduced a technique for forming large diameter-to-thickness ratio tubes by nesting them into outer tubes using controlled bending deformation laws and composite bending deformation. Cui et al. [20] developed a strategy to mitigate springback in hydroformed tubular parts, enhancing their dimensional accuracy. Through experimental investigation, they explored the relationship between diameter change, springback, and internal pressure during hydroforming. This analysis revealed that controlling internal pressure is crucial for achieving optimal dimensional accuracy. The highest accuracy is attained when the expansion of the mold cavity precisely matches the rebound of the tubular part. In the liquid-filled hydraulic forming process [21], the liquid inside the tube lumen serves a supporting role and does not need to be under high pressure. This avoids the sealing problems caused by ultra-high liquid pressure in internal high-pressure forming and reduces the cost of sealing both ends of the tube with large hydraulic equipment. Since the tube is only subjected to hydraulic pressure, it experiences a uniaxial stress state dominated by mechanical forces. Building on this, the mechanical force generated by the movement of the mold or rigid mechanism, combined with the hydraulic loading path, can control the stress state of the material in the tube. This significantly improves the forming limit of the material, preventing part rupture due to over-thinning [22,23,24].

In this research, the bending deformation analysis of aluminum alloy double-lumen tubes was examined using the liquid-filled bending method. The study explored the bending deformation behavior of aluminum alloy double-lumen tubes under the theoretical framework of liquid-filled bending, considering various parameters of double-lumen tubes. By applying the deformation theory of aluminum alloy double-lumen tubes, the formation mechanisms of inner and outer defects in double-lumen tubes were analyzed, providing theoretical guidance for the liquid-filled bending of double-lumen tubes. Through a combination of experimental research and numerical simulation, the effects of internal support pressure, bending radius, and tube wall thickness on the bending behavior of liquid-filled double-lumen tubes were elucidated.

## 2. Liquid-Filled Bending Principle for Reinforced Profiles

Tube liquid-filled bending and forming technology [25] combines tube bending and pressing technology with hydraulic forming, using high-pressure liquid as the filling medium [24]. This composite process not only retains the characteristics of traditional bending and pressing forming but also incorporates the advantages of hydraulic forming, effectively reducing forming defects during tube bending and deformation. While mature tube liquid-filled forming techniques primarily focus on punching hydraulic bending and punching liquid filler push bending, research on aluminum alloy multi-lumen tube liquid-filled bending remains limited. This paper explores the application of liquid-filled bending technology to aluminum alloy multi-lumen tube bending deformation testing.

**Figure 1:**
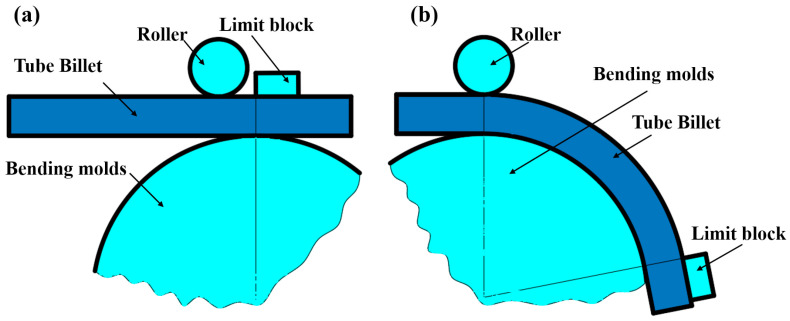
Principle of liquid-filled bend forming: (**a**) pre-deformation; (**b**) post-deformation.

Liquid-filled bypass bending is a plastic forming technology that utilizes the closing force of the mold or the bending force of the bending die to shape an aluminum alloy double-lumen tube into the desired fitting configuration, while the internal pressure of the liquid within the sealed tube provides support, as illustrated in Figure 1.

Liquid-filled bending enables the achievement of variable curvature tube bending deformation, which is challenging to accomplish with conventional tube bending methods, particularly when processing non-circular cross-sectional tubes. The liquid-filled bending process effectively addresses molding defects by applying liquid pressure. Lin et al. [26] achieved low-pressure forming of 6063 aluminum alloy by pressing the tube cross-section from a prefabricated ellipse to a square shape using the mechanical force provided by the mold closing process supported by a lower internal liquid pressure. In this paper, an internal liquid-filled bending and forming method for ribbed aluminum alloy double-lumen tubes is proposed on this basis. However, the mold or bending die cannot always provide uniform constraints on the tube, resulting in some surfaces being in a free state during the forming process. Consequently, the tube billet is subjected to complex stresses, necessitating the investigation of typical cross-sectional distortion degrees on both the inner and outer surfaces of the tube. This research aims to identify a key method for improving the bending accuracy of the internal and external surfaces during the bending process.

## 3. Experimental

### 3.1. Experimental Materials

**Figure 2:**
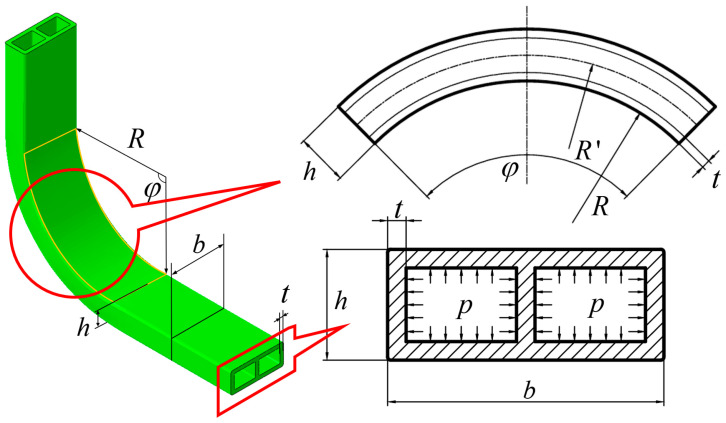
Shape and dimensions of aluminum alloy double-lumen tube bend specimen.

The experimental study employed 6063-T5 aluminum alloy double-lumen tubes, whose chemical composition is presented in Table 1. The geometric and size specifications of the bent tube are illustrated in Figure 2, while the mechanical properties are summarized in Table 2. The tube had a length of 250 mm (with a measured value of 250 ± 0.5 mm), a billet cross-sectional length of 30 mm (with a measured value of 30 ± 0.1 mm), a height of 12 mm (with a measured value of 12 ± 0.1 mm), and a wall thickness of 2 mm (with a measured value of 2.0 ± 0.1 mm). The billet’s axial direction length was 250 mm, with an equivalent radius of 13.36 mm, a single-cavity equivalent radius of 8.9 mm, a diameter-to-thickness ratio (*λ* = *d*/*t*) of 13.36, and a single-cavity diameter-to-thickness ratio of 8.9.

### 3.2. Experimental Devices

**Figure 3:**
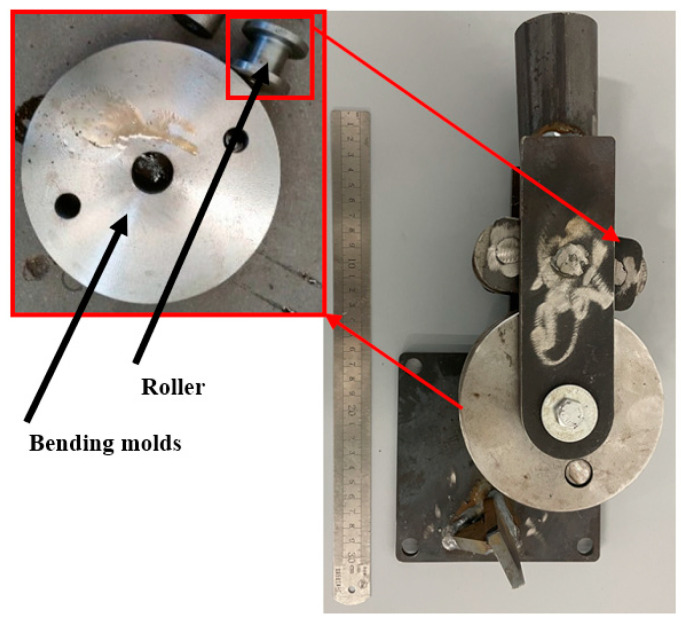
Wrap-around forming mold.

The liquid-filled tube bending and forming process involves the rotation of the roller around the bending mold, where the restriction block constrains the tube to bend around the mold, as illustrated in Figure 3. The primary components of the mold include the bending mold and roller. The mold was designed to conform to the inner wall cross-sectional shape of the tube billet, with a groove-shaped steel plate on the side of the tube wall that provides a restrictive effect.

**Figure 4:**
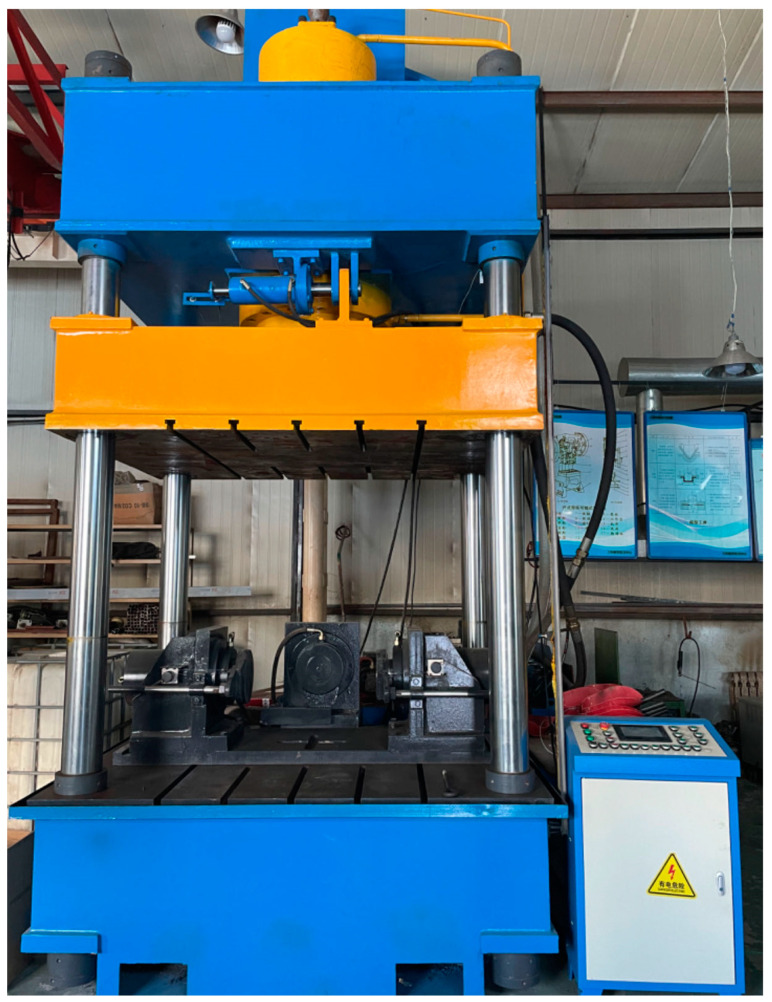
T-slot operating platform.

Additionally, a T-slot operating table was equipped to install the bending mold, as depicted in Figure 4. The T-slot operating table was securely connected to the bending mold via a fixed connection using a pressure plate.

**Figure 5:**
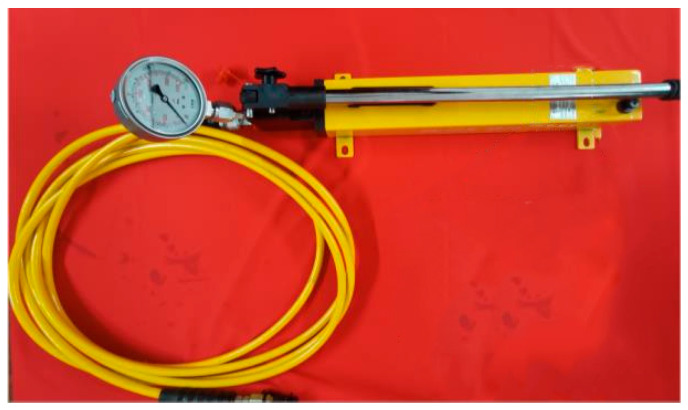
Manual oil pump.

Due to the low pressure of the liquid in the tube, a manual oil pump was employed to fill the tube and apply liquid pressure. The tank had a capacity of 3 L and was rated for ISO 46 anti-wear hydraulic oil. The oil outlet was connected to a hydraulic oil tube, which was equipped with a quick-disconnect connector at one end, allowing for rapid connection to the billet nozzle to form a closed loop. The manual oil pump was connected to a hydraulic table, which was used to read the specific value of the pressure in the tube, as illustrated in Figure 5.

### 3.3. Experimental Program

Variations in process parameters can significantly impact the forming quality of liquid-filled bending components. Therefore, this study investigated the effects of these parameters on the bending quality of double-lumen tubes by adjusting them. The primary parameters influencing the aluminum alloy double-lumen tube bending process were support pressure, bending radius of the billet, and tube wall thickness. The upper surface of the tube lumen after bending and deformation of the tubing was the inner side of the bend, and the lower surface was the outer side of the bend. The internal support pressure affected the deformation of the tube wall during the bending process; the bending radius influenced the accumulation of material per unit angle and the degree of stretching; and the forming difficulty of billets with different wall thicknesses varied, making the bending and forming parts more prone to defects.

To investigate the effects of these parameters, this study focused on the cross-sectional distortion, stress distribution, and wall thickness distribution in the bending region of the billet. The research program was divided into three groups: Group 1: Investigated the effect of support pressure on the bending of the billet, with a constant bending radius of R = 50 mm and wall thickness of t = 2 mm, using six different internal pressure values. Group 2: Examined the effect of bending radius on the molding process, with a constant internal pressure of *p* = 5 MPa and wall thickness of t = 2 mm, using three different bending radii. Group 3: Studied the effect of wall thickness on the molding process, with a constant internal pressure of *p* = 5 MPa and bending radius of R = 50 mm, using six different wall thickness values. The test pieces were subjected to bending through a 90° angle using the bending mold under various test conditions. This allowed for the observation of deformation under different process parameters, as summarized in Table 3. In the 90 degree bending test, a specimen was considered qualified only if it met the following requirements: the bending forming angle error was limited to ±1.5°, and the tube wall depression did not exceed 0.5 mm.

## 4. Theoretical Analysis

### 4.1. Force Analysis

During the bending of a double-lumen tube filled with liquid, forces are exerted by the bending mold, necessitating a force analysis of the bending process. The coordinate system XOY is defined with the origin located at the center of the tube where the bending part and the straight section part intersect. The X-axis is parallel to the prism of the straight section tube. During the bending process, tube fittings encounter various forces across distinct states. The force $F_{1}$, applied by the roller in the direction of the bending radius, induces bending deformation in the pipe fittings, pressing them against the bending mold. Concurrently, the bending mold exerts a distributed force $F_{2}$, while the billet’s end faces a supporting counterforce $F_{3}$ from the restriction block, ensuring the billet remains stationary within the mold throughout the bending process. Throughout this process, the combined actions of $F_{1}$, $F_{2}$, and $F_{3}$ are constant. Specifically, the distribution of $F_{2}$ across the contact areas between the tube bending part and the mold is critical. Directly formulating force equilibrium equations for this scenario results in a statically indeterminate problem, necessitating numerical simulation for analysis, as detailed in Section 5 of this manuscript. For practical engineering applications, simplifying $F_{2}$ is essential to derive analytical equations. Therefore, $F_{2}$ is assumed to be a concentrated force located at the midpoint of the contact area between the tube wall and the roller. This assumption allows for considering the interplay of $F_{1}$, $F_{2}$, and $F_{3}$ as three equilibrium forces acting simultaneously to bend the tubing at any given moment.

**Figure 6:**
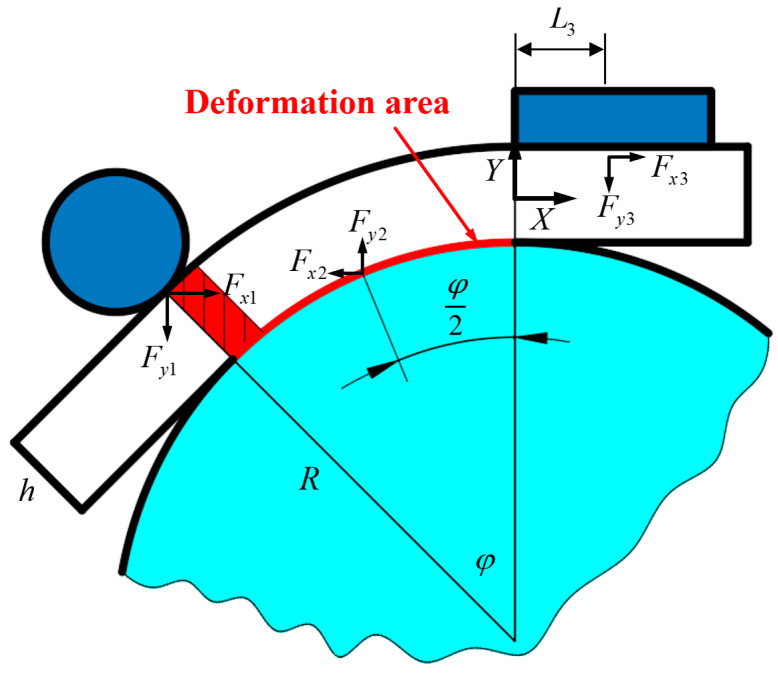
Overall force analysis of tube fittings.

The forces acting on the billet at the rollers, the bending die, and the limiting block are decomposed into two forces in the x-direction and the y-direction. Markings x and y in the lower right corner represent parallelism with the X-axis and the Y-axis. $F_{1}$ is decomposed into $F_{x1}$ and $F_{y1}$, $F_{2}$ is decomposed into $F_{x2}$ and $F_{y2}$, and $F_{3}$ is decomposed into $F_{x3}$ and $F_{y3}$. The static equilibrium equations are used to deduce the magnitude of the force at each point. The force analysis diagram of tube billet winding and bending forming is shown in Figure 6.

The forces on the billet during bending and forming are analyzed and the following static equilibrium equations are derived:

(1) $$\left\{\begin{array}{l} F_{x1}-F_{x2}+F_{x3}=0\\ F_{x2}L_{x2}+F_{y2}L_{y2}-F_{x1}L_{x1}-F_{y1}L_{y1}+F_{x3}\frac{h}{2}+F_{y3}L_{3}=0\\ F_{y2}-F_{y1}-F_{y3}=0 \end{array}\right.$$

The expressions $L_{x1}$, $L_{y1}$, $L_{x2}$, and $L_{y2}$ are introduced for the convenience of narration to refer to the perpendicular distances from the point of action of their forces to the coordinate axes, as follows:

(2) $$\left\{\begin{array}{l} L_{x1}=R\left(1-\cos\varphi \right)-h\cos\varphi +\frac{h}{2}\\ L_{y1}=\left(R+h\right)\sin\varphi \\ L_{x2}=R\left(1-\cos\frac{\varphi }{2}\right)+\frac{h}{2}\\ L_{y2}=R\sin\frac{\varphi }{2} \end{array}\right.$$

where the two components of $F_{1}$, $F_{x1}$ and $F_{y1}$, and the two components of $F_{2}$, $F_{x2}$ and $F_{y2}$, in their relations are, respectively, as follows:

(3) $$\frac{F_{x1}}{F_{y1}}=\tan\varphi$$

(4) $$\frac{F_{x2}}{F_{y2}}=\tan\frac{\varphi }{2}$$

**Figure 7:**
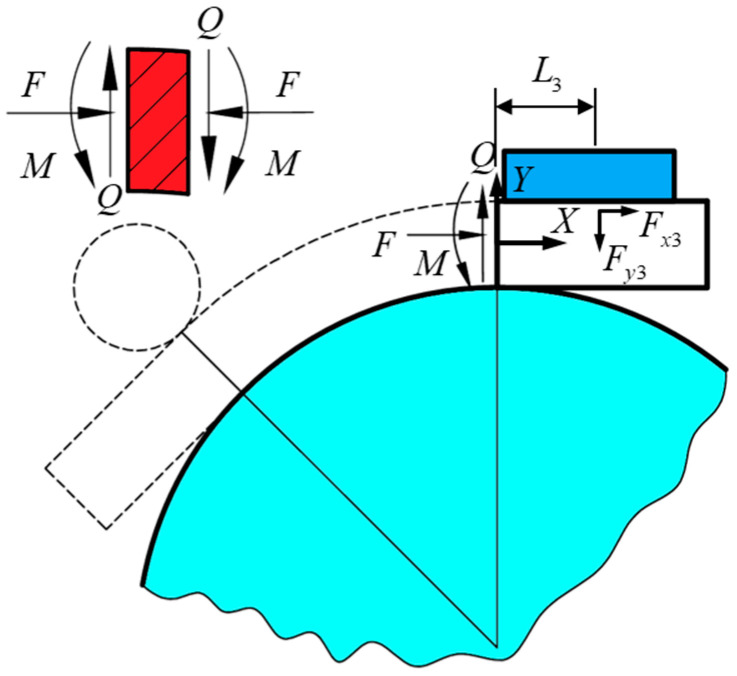
Local force analysis of tube fittings.

In Equation (1), $F_{x3}$ is the static friction between the limiting block and the tube billet, and its magnitude is the sum of the external forces in the direction of friction force when no relative sliding occurs. In order to simplify the problem, the force analysis of the formed tube is carried out using the local method, as shown in Figure 7; the tube is dissected at the right boundary of the deformation zone; and the right boundary of the deformation zone is subjected to the tangential pressure *F*, shear force *Q*, and bending moment *M*. A force analysis is carried out in the localized dissected region, which can be obtained as

(5) $$\left\{\begin{array}{l} Q-F_{y3}=0\\ F_{x3}\cdot \frac{h}{2}+F_{y3}\cdot L_{3}+M=0\\ F=F_{x3} \end{array}\right.$$

The expressions for the bending force $F_{1}$ of the roller on the tubing are obtained by combining Equations (1)–(5):

(6) $$F_{1}=-F\cdot \frac{L_{x2}\tan\frac{\varphi }{2}+L_{y2}+L_{3}+\frac{h}{2}\tan\frac{\varphi }{2}}{A-B}$$

In liquid-filled tube bending and forming, the internal pressure applied by the sealed liquid medium significantly influences the bending and forming quality. When the internal pressure is too small, the billet is prone to bending and cross-sectional deformation defects due to the combined effects of bending, internal pressure, and mold compression forces. In contrast, when the internal pressure is too large, the tube wall is subjected to excessive stress, exceeding the yield strength of the material, leading to tensile plastic deformation, distension of the billet, and cross-sectional deformities. Furthermore, the tube wall may be squeezed between the molds, resulting in flying edge defects, or stretched, leading to cracking defects. Therefore, it is essential to determine the optimal internal liquid limit support pressure through force analysis.

**Figure 8:**
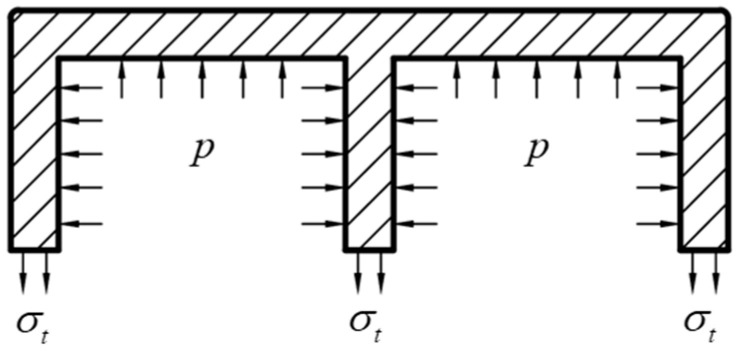
Stresses on the cross-section of a double-lumen tube.

Let $p_{s}$ denote the limit support pressure, which is the maximum allowable value of the liquid pressure inside the tube to ensure that it does not expand. The liquid inside the tube provides support to both the inner and outer walls as well as the side walls. Under the same internal pressure, the ultimate support pressure at different locations of the tube wall is not the same at which expansion occurs. According to the stress on the cross-section in Figure 8, the ultimate support internal pressure of different tube walls is solved. Let $\sigma_{t}=\sigma_{s}$, analyze the billet cross-section length, the compression length is (b−3t)/2, then the limiting support internal pressure of the tube wall of the billet cross-section length is as follows:

(7) $$p_{s}=\frac{4t}{b-3t}\sigma_{s}$$

The shorter side (lateral tube wall) is analyzed for the force, and the compression length is h−2t; then, the ultimate support internal pressure in the side wall of the tube is as follows:

(8) $$p_{s}=\frac{2t}{h-2t}\sigma_{s}$$

where $p_{s}$ is the ultimate support internal pressure, $\sigma_{s}$ is the material yield strength, b is the billet section length, h is the billet section width, and t is the billet wall thickness.

**Figure 9:**
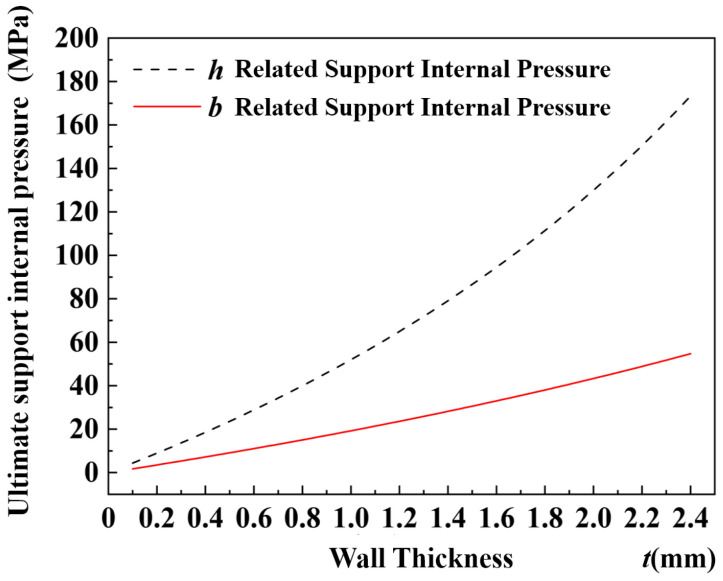
Ultimate support internal pressure for different wall thicknesses.

For the 6063-T5 aluminum alloy material, the radial stress $\sigma_{s}$ was 130 MPa. The tube had a width *b* of 30 mm and a height *h* of 12 mm. The relationship curves between the ultimate internal pressure of the support and the wall thickness can be obtained for the tube wall at different locations, as shown in Figure 9. The two curves in Figure 9 show the variation in ultimate internal pressure exerted on the length and width of the tubing cross-section under different wall thicknesses. It can be observed from the curves that, for the cross-section of the tube, the ultimate pressure along the width was greater than the pressure along the length. Consequently, the pressure limit for the tube should be determined based on the pressure limit measured at the length of the cross-section, in order to ascertain the pressure range within the tube when it is filled with liquid.

### 4.2. Stress Analysis

To determine the stresses at each characteristic point, namely, the principal stresses $\sigma_{\theta }$, $\sigma_{r}$, and $\sigma_{L}$ in the three directions, it is necessary to solve three separate equations independently. This involves analyzing the microelement body at any position on the bending arc segment and listing the differential equilibrium equations, deformation compatibility equations, and constitutive equations based on various equilibrium conditions and elastic-plasticity theory.

**Figure 10:**
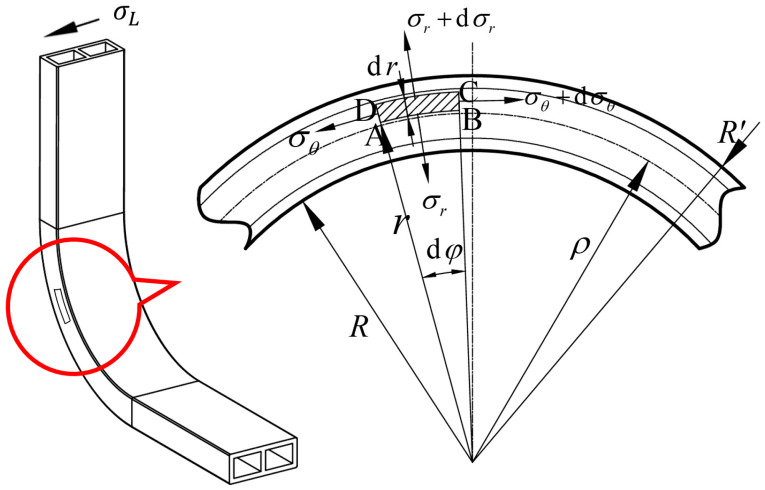
Force analysis of microelement body in the deformation zone of rectangular cross-section tube.

A fan-ring-shaped microelement body ABCD is selected at a specific position on the wall within the bending and forming zone of the double-lumen tube, and its force situation is analyzed as shown in Figure 10. The symbols in the figure are defined as follows: The inner and outer arcs of the fan-ring microelement body are concentric with the bending arc segment. The inner radius of the fan-ring is *r*. The height of the fan-ring is *dr*. The maximum radius of the fan-ring is *r* + *dr*. The fan-ring circumferential angle is *dφ*. The width is approximately *db*. The strain-neutral layer has a radius of *ρ*. The inner radius of the bend of the deformation zone is *R*. The outermost radius is *R*′. The fan-ring is subject to tangential stresses on the left end, denoted by $\sigma_{\theta }$, and on the right side, denoted by $\sigma_{\theta }+d\sigma_{\theta }$. The microelement is also subjected to radial stresses along the radius pointing towards the center of the circle, denoted by $\sigma_{r}$, and radial stresses on the outer arc of the circle back to the center of the circle, denoted by $\sigma_{r}+d\sigma_{r}$. The Poisson’s ratio of the material is *μ*.

Since the double-lumen billet undergoes gradual and progressive bending deformation, any point in the deformation zone is in equilibrium. The chosen microelement body is assumed to be in a planar state, neglecting the influence of circumferential stress $\sigma_{L}$. This simplification implies that the width of the workpiece remains constant throughout the deformation process. Within the plane where the microelement is located, the microelement is subjected to only two principal stresses: the tangential stress $\sigma_{\theta }$ and the radial stress $\sigma_{r}$. To simplify the calculations, the effect of friction is disregarded in the analysis.

The radial force on the AB surface pointing in the direction of the center of the circle and the radial force on the CD surface away from the center of the circle is as follows:

(9) $$F_{AB}=r\sigma_{r}d\theta db$$

(10) $$F_{CD}=\left(\sigma_{r}+d\sigma_{r}\right)(r+dr)d\theta db$$

The radial component of the tangential stress on the AD face and the radial component of the tangential stress on the BC face is as follows:

(11) $$F_{AB}=\sigma_{\theta }\sin\frac{d\varphi }{2}drdb\approx \sigma_{\theta }\frac{d\varphi }{2}drdb$$

(12) $$F_{BC}=(\sigma_{\theta }+d\sigma_{\theta })\sin\frac{d\varphi }{2}drdb\approx (\sigma_{\theta }+d\sigma_{\theta })\frac{d\varphi }{2}drdb$$

Any point at any position in the deformation region is in equilibrium, and the equilibrium equations are made in the radial direction of the microtome so that the algebraic sums of $F_{AB}$, $F_{CD}$, $F_{AD}$, and $F_{BC}$ of Equations (9)–(12) are zero, neglecting the higher-order infinitesimal quantities $\frac{1}{2}d\sigma_{\theta }dr$ and $-d\sigma_{r}dr$, and collapsing the obtained afterward as

(13) $$\frac{\sigma_{\theta }-\sigma_{r}}{r}=\frac{d\sigma_{r}}{dr}$$

The inner and outer walls of the tube billet are constrained circumferentially by the inner wall of the bending mold, which is in a plane stress–strain state. As a result, there is no length change in the circumferential direction, and the circumferential strain is zero, $\varepsilon_{L}=0$. During the elastic deformation stage, where plastic deformation does not occur, there is a slight change in volume due to elastic deformation. However, when the material enters the plastic deformation stage, the change in volume caused by elastic deformation is negligible compared to that caused by plastic deformation. Therefore, the change in volume due to elastic deformation can be ignored. Furthermore, since the plastic deformation is relatively small, the bending deformation of the billet approximately complies with the law of volume invariance of plastic deformation of materials.

Substituting $\varepsilon_{L}=0$ for the law of volume invariance for plastic deformation of materials $\varepsilon_{\theta }+\varepsilon_{r}+\varepsilon_{L}=0$, it is obtained as

(14) $$\varepsilon_{\theta }=-\varepsilon_{r}$$

The circumferential strain is zero, and substituting $\varepsilon_{L}=0$ into the full volume theory equation $\varepsilon_{L}=\frac{\bar{\varepsilon }}{\bar{\sigma }}\left[\sigma_{L}-\frac{1}{2}(\sigma_{\theta }+\sigma_{r})\right]$, is as follows:

(15) $$\sigma_{L}=\frac{1}{2}(\sigma_{\theta }+\sigma_{r})$$

Substituting $\sigma_{L}$ in Equation (15) into the equivalent force expression $\bar{\sigma }=\frac{1}{\sqrt{2}}\sqrt{(\sigma_{\theta }-\sigma_{r}{)}^{2}+(\sigma_{\theta }-\sigma_{L}{)}^{2}+(\sigma_{L}-\sigma_{r}{)}^{2}}$, and replacing $\sigma_{L}$ with $\sigma_{\theta }$ and $\sigma_{r}$, is obtained by collation as follows:

(16) $$\bar{\sigma }=\frac{\sqrt{3}}{2}\left|\sigma_{\theta }-\sigma_{r}\right|$$

Substituting $\varepsilon_{L}=0$ and $\varepsilon_{\theta }=-\varepsilon_{r}$, into the Mises equivalent effect transformation expression $\bar{\varepsilon }=\frac{\sqrt{2}}{2(1+\mu )}\sqrt{(\varepsilon_{\theta }-\varepsilon_{r}{)}^{2}+(\varepsilon_{\theta }-\varepsilon_{L}{)}^{2}+(\varepsilon_{L}-\varepsilon_{r}{)}^{2}}$, in which μ is taken to be 0.5 due to plastic deformation of the tube billet, is collapsed and obtained as follows:

(17) $$\bar{\varepsilon }=\frac{2}{\sqrt{3}}\left|\varepsilon_{\theta }\right|$$

$\bar{\sigma }=K\cdot {\bar{\varepsilon }}^{n}$ is the true stress–strain expression, which can be obtained by substituting and collapsing the $\bar{\sigma }$ solved in Equation (9) and the $\bar{\varepsilon }$ of Equation (10):

(18) $$\frac{\sqrt{3}}{2}\left|\sigma_{\theta }-\sigma_{r}\right|=K\cdot {\left(\frac{2}{\sqrt{3}}\left|\varepsilon_{\theta }\right|\right)}^{n}$$

In the above formula, K is the strength coefficient, and *n* is the strain hardening index.

The true strain $\varepsilon =\int_{l_{0}}^{l_{n}}\frac{dl}{l}=\ln\frac{l_{n}}{l_{0}}$, whereas in this equation, $l_{n}$ is rdφ, and $l_{0}$ is ρdφ, which can be obtained by substituting it into

(19) $$\varepsilon_{\theta }=\ln\frac{l_{n}}{l_{0}}=\ln\frac{rd\varphi }{\rho d\varphi }=\ln\frac{r}{\rho }$$

Substituting Equation (19) into Equation (18) yields

(20) $$\left|\sigma_{\theta }-\sigma_{r}\right|=K\cdot {\left(\frac{2}{\sqrt{3}}\right)}^{n+1}{\left(\pm \ln\frac{r}{\rho }\right)}^{n}$$

The sign of “$\pm \ln\frac{r}{\rho }$” in the above equation depends on the location, and it is taken to be positive when the feature point is on the outer tube wall and negative when it is on the inner tube wall.

Substituting Equation (20) into Equation (13) and substituting the absolute values equivalently gives

(21) $$\frac{d\sigma_{r}}{dr}=\pm \frac{K\cdot {\left(\frac{2}{\sqrt{3}}\right)}^{n+1}{\left(\pm \ln\frac{r}{\rho }\right)}^{n}}{r}$$

The equation is integrated to gain the following:

(22) $$\sigma_{r}=\frac{K}{n+1}{\left(\frac{2}{\sqrt{3}}\right)}^{n+1}{\left(\pm \ln\frac{r}{\rho }\right)}^{n+1}+C_{1,2}$$

The inner and outer walls of a double-lumen billet tend to zero stress near the outer surface of the material, and there is no stress on the outermost surface of the material, i.e., $\sigma_{r}=0$ for $r=R^{\prime }$ and r=R. For the outer walls,

(23) $$\sigma_{R^{\prime }}=\frac{K}{n+1}\cdot {\left(\frac{2}{\sqrt{3}}\right)}^{n+1}{\left(\ln\frac{R^{\prime }}{\rho }\right)}^{n+1}+C_{1}=0$$

For the internal tube wall,

(24) $$\sigma_{R}=\frac{K}{n+1}\cdot {\left(\frac{2}{\sqrt{3}}\right)}^{n+1}{\left(-\ln\frac{R}{\rho }\right)}^{n+1}+C_{2}=0$$

Calculations are available as follows:

(25) $$C_{1}=-\frac{K}{n+1}{\left(\frac{2}{\sqrt{3}}\right)}^{n+1}{\left(\ln\frac{R^{\prime }}{\rho }\right)}^{n+1}$$

(26) $$C_{2}=-\frac{K}{n+1}{\left(\frac{2}{\sqrt{3}}\right)}^{n+1}{\left(-\ln\frac{R}{\rho }\right)}^{n+1}$$

The expressions of two constant terms, $C_{1}$ and $C_{2}$, in Equation (22) are obtained to acquire the expression of $\sigma_{r}$. Substituting the expression of $\sigma_{r}$ into Equation (20) and Equation (15), respectively, the specific expressions of $\sigma_{L}$ and $\sigma_{\theta }$ are obtained, and thus the analytical equation of the stress on the outer wall of the tube billet can be obtained as

(27) $$\left\{\begin{array}{l} \sigma_{r}=\frac{K}{n+1}{\left(\frac{2}{\sqrt{3}}\right)}^{n+1}\left[{\left(\ln\frac{r}{\rho }\right)}^{n+1}-{\left(\ln\frac{R^{\prime }}{\rho }\right)}^{n+1}\right]\\ \sigma_{\theta }=K\cdot {\left(\frac{2}{\sqrt{3}}\right)}^{n+1}\left\{\frac{1}{n+1}\left[{\left(\ln\frac{r}{\rho }\right)}^{n+1}-{\left(\ln\frac{R^{\prime }}{\rho }\right)}^{n+1}\right]+{\left(\ln\frac{r}{\rho }\right)}^{n}\right\}\\ \sigma_{L}=\frac{K}{2}\cdot {\left(\frac{2}{\sqrt{3}}\right)}^{n+1}\left\{\frac{2}{n+1}\left[{\left(\ln\frac{r}{\rho }\right)}^{n+1}-{\left(\ln\frac{R^{\prime }}{\rho }\right)}^{n+1}\right]+{\left(\ln\frac{r}{\rho }\right)}^{n}\right\} \end{array}\right.$$

The analytical equation for the stress in the internal tube wall of the billet are obtained as

(28) $$\left\{\begin{array}{l} \sigma_{r}=\frac{K}{n+1}{\left(\frac{2}{\sqrt{3}}\right)}^{n+1}\left[{\left(-\ln\frac{r}{\rho }\right)}^{n+1}-{\left(-\ln\frac{R}{\rho }\right)}^{n+1}\right]\\ \sigma_{\theta }=K\cdot {\left(\frac{2}{\sqrt{3}}\right)}^{n+1}\left\{\frac{1}{n+1}\left[{\left(-\ln\frac{r}{\rho }\right)}^{n+1}-{\left(-\ln\frac{R}{\rho }\right)}^{n+1}\right]-{\left(-\ln\frac{r}{\rho }\right)}^{n}\right\}\\ \sigma_{L}=\frac{K}{2}\cdot {\left(\frac{2}{\sqrt{3}}\right)}^{n+1}\left\{\frac{2}{n+1}\left[{\left(-\ln\frac{r}{\rho }\right)}^{n+1}-{\left(-\ln\frac{R}{\rho }\right)}^{n+1}\right]-{\left(-\ln\frac{r}{\rho }\right)}^{n}\right\} \end{array}\right.$$

When deriving the stress–strain analytical equation for the lateral tube wall and the fascia plate, it is essential to consider that the tube wall on the outer side of the lateral tube wall bending is decoupled from the leaning, whereas on the inner side, it is restricted by the bending mode. As a result, the lateral tube wall is not constrained in the circumferential direction, $\sigma_{L}=0$. On the outer side of the bending, $\sigma_{\theta }>0$ and $\sigma_{r}<0$, indicating that $\sigma_{\theta }>\sigma_{L}>\sigma_{r}$. The tangential stress on the inside of the lateral tube wall bending is a compressive stress, $\sigma_{\theta }>0$ and $\sigma_{r}<0$. The dominant stress in the bending and forming process is the tangential stress $\sigma_{\theta }$, and its absolute value is greater than the absolute value of $\sigma_{r}$. Therefore, the relationship between the stresses is $\sigma_{r}<\sigma_{L}=0<\sigma_{\theta }$. Given that the stress state of the stiffener plate is identical to that of the lateral tube wall, it can be assumed that the analytical stress equation developed for the lateral tube wall is applicable to the stiffener plate as well. According to $\sigma_{r}<\sigma_{L}=0<\sigma_{\theta }$, substituting $\sigma_{s}=\frac{1}{\beta }\sqrt{(\sigma_{\theta }-\sigma_{r}{)}^{2}+(\sigma_{\theta }-\sigma_{L}{)}^{2}+(\sigma_{L}-\sigma_{r}{)}^{2}}$ can be obtained:

(29) $$\sigma_{\theta }-\sigma_{r}=\beta \sigma_{s}$$

The above formula β is the intermediate principal stress influence coefficient, generally taking 1~1.155.

Substituting Equation (29) into Equation (13) gives

(30) $$\frac{d\sigma_{r}}{dr}=\frac{\beta \sigma_{s}}{r}$$

Integrating both sides of the equation obtains $\sigma_{r}$ as

(31) $$\sigma_{r}=\beta \sigma_{s}\cdot \ln r+C_{3}$$

The radial stress $\sigma_{r}$ tends to zero in the inner and outer walls of the double-lumen billet near the outer surface of the material, and there is no stress distribution on the outermost surface, $\sigma_{r}=0$, when $r=R^{\prime }$, and there is when r=R, $\sigma_{r}=0$, as follows:

(32) $$\beta \sigma_{s}\cdot \ln R^{\prime }+C_{3}=0$$

(33) $$\beta \sigma_{s}\cdot \ln R+C_{4}=0$$

Calculated to gain as follows:

(34) $$C_{3}=-\beta \sigma_{s}\cdot \ln R^{\prime }$$

(35) $$C_{4}=-\beta \sigma_{s}\cdot \ln R$$

The constant term in the expression of $\sigma_{r}$ is obtained; the expression of $\sigma_{r}$ is substituted into (29); and the collation is obtained, thus obtaining the expression of $\sigma_{\theta }$, so the analytical equation of the stresses at the outer side of the lateral tube wall bending and at the outer side of the fascia bending is

(36) $$\left\{\begin{array}{l} \sigma_{r}=\beta \sigma_{s}\cdot \ln\frac{r}{R^{\prime }}\\ \sigma_{\theta }=\beta \sigma_{s}\cdot \left(1+\ln\frac{r}{R^{\prime }}\right)\\ \sigma_{L}=0 \end{array}\right.$$

For the inside of the bend, the expression is

(37) $$\left\{\begin{array}{l} \sigma_{r}=-\beta \sigma_{s}\cdot \ln\frac{r}{R}\\ \sigma_{\theta }=-\beta \sigma_{s}\cdot \left(1+\ln\frac{r}{R}\right)\\ \sigma_{L}=0 \end{array}\right.$$

Equations (27), (28), (36) and (37) are stress expressions for six characteristic points in four regions of the billet.

## 5. Finite Element Model

**Figure 11:**
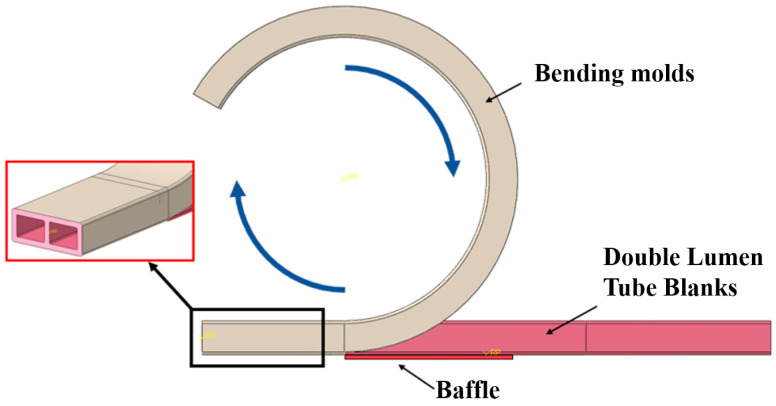
Simplified geometric model of the bending mold.

**Figure 12:**
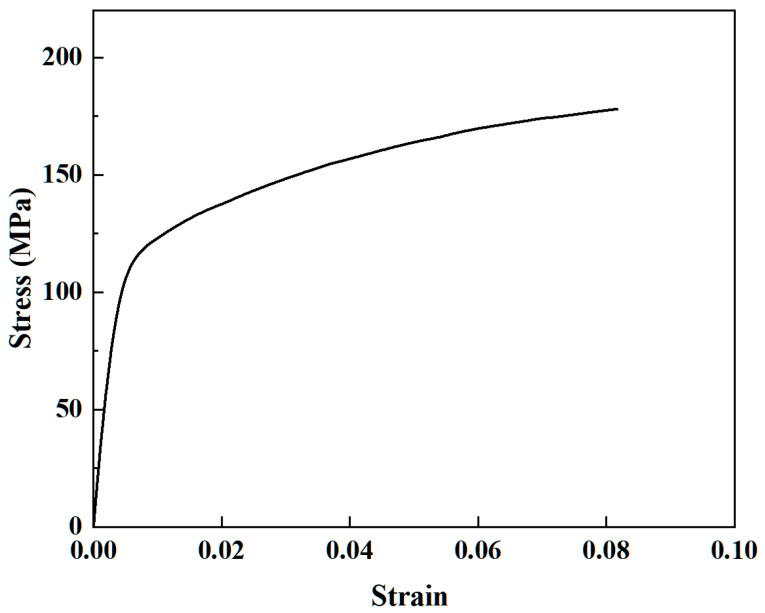
Stress–strain curve of 6063-T5.

Based on the principle of liquid-filled bending and forming of double-lumen tubes, a geometric model of the bending and winding mold has been established. This model maintains the same contact and boundary conditions as those of the experimental mold, thereby constructing a simulation platform that replicates the experimental environment. To minimize simulation time and ensure that the simulation results are closely aligned with the experimental outcomes, the finite element model must be simplified as much as possible. For the finite element model design, the bending mold and billet share the same rectangular cross-sectional shape, with the tube surface connected to a segment of the bending groove steel-shaped surface. The billet geometry mirrors the experimental billet, without welded seals on either side. Design a baffle plate close to the billet instead of the roller, define the baffle plate is completely fixed, the bending mold around the center of the circular motion, the rotation angle of 90°, converted to radian system for −1.571 radians. Bending of the billet is carried out by restraining the left end of the billet through the bending mold cavity; the bending mold rotation drives the billet to rotate, and due to the blocking effect of the baffle plate, the double-lumen tube model is pressed against the baffle plate to undergo bending deformation. The location of the baffle plate is the connection between the rectangular tube surface of the bending mold and the shaped surface of the bending channel steel, as shown in Figure 11. The numerical simulation of the tube was made through the dynamic explicit module of ABAQUS [27], and the yield stress of the model was set to 130 MPa according to the stress–strain curve in Figure 12.

**Figure 13:**
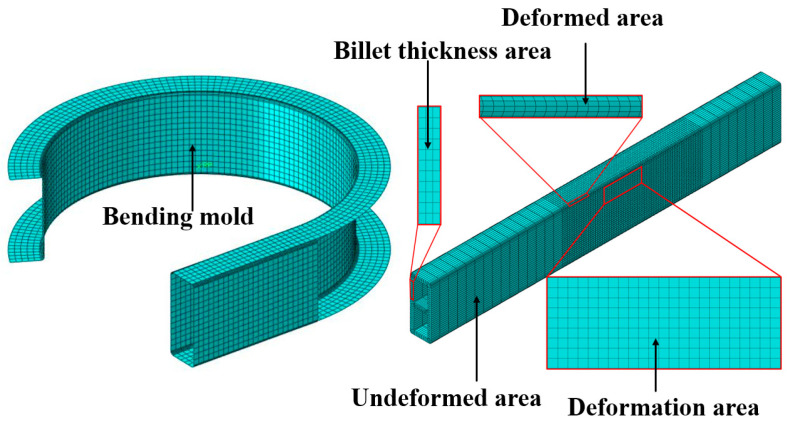
Finite element model meshing results.

**Figure 14:**
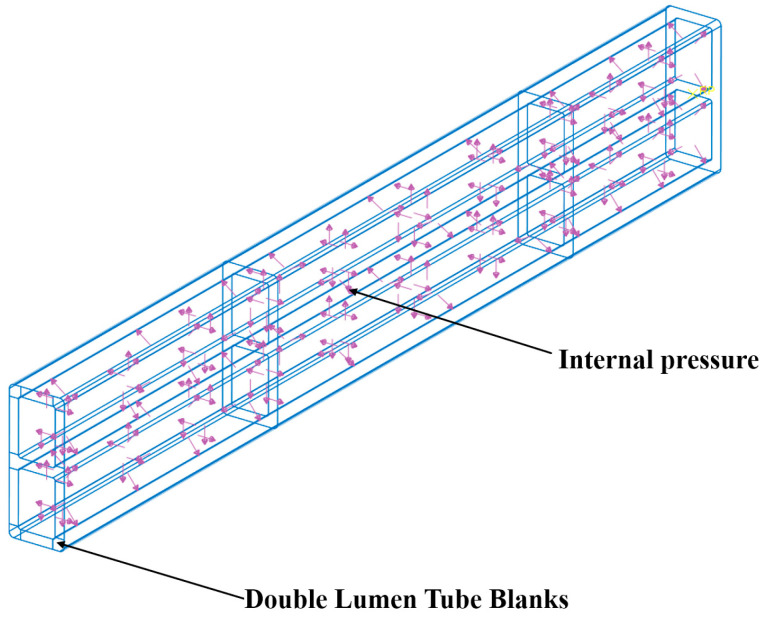
Load applied to the inside of a double-lumen tube.

In order to ensure the accuracy of the calculation results, the type of the tube billet was set as a 3D deformable solid, the cell type was set as an eight-node linear hexahedral cell (C3D8R), and the mesh cell was drawn using the hexahedral sweep progression algorithm; the bending mode was a discrete rigid shell cell, set as a four-node 3D bilinear rigid quadrilateral (R3D4), and the mesh cell was drawn using the tetrahedral free progression algorithm. Moreover, the baffle plate was set as a rigid body. To ensure both computational efficiency and simulation accuracy, a meshing strategy was employed. The deformation zone was discretized with a node density of 1 node/1 mm, while the undeformed zone used a coarser density of 1 node/5 mm. In the billet’s thickness direction, a node density of 1 node/1 mm was maintained. This resulted in a total of 37,280 elements for the billet. The bending mold was meshed with a node density of 1 node/2 mm, leading to 9510 elements. As illustrated in Figure 13, mesh sensitivity analysis confirmed that this mesh configuration adequately captured the structural response of the component. The coefficient of friction between the billet and the die was determined through a friction basis experiment. The mean value of the coefficient of friction between the aluminum tube and the die was found to be 0.3. We defined the contact friction coefficient between the bending die and the billet, the baffle plate and the bending die as 0.3, with the contact type being surface-to-surface contact. The pressure on the inner wall surface of the double-lumen tube billet was defined as a single experimental design value. This pressure load was applied inside the double-lumen tube billet, as shown in Figure 14.

**Figure 15:**
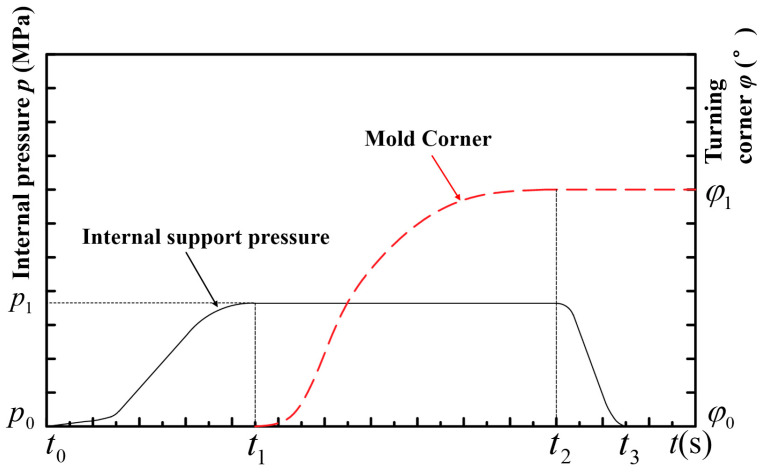
Liquid-filled bending loading curve.

During the liquid-filled bending process, the first step involved filling the tube with hydraulic oil and pressurizing it. Once the internal pressure reached *p*_1_, the pressure application was stopped and maintained at this value. After completing the pressure loading, the pry bar was activated, which drove the movement of the rollers, pushing the tube billet against the bending mold until the angle of rotation reached *φ*_1_, at which point the movement was stopped. Finally, the internal pressure was released, completing the bending and deformation process. The internal pressure loading curve is illustrated in Figure 15.

## 6. Results Analysis

### 6.1. Validation of FEA Model

**Figure 16:**
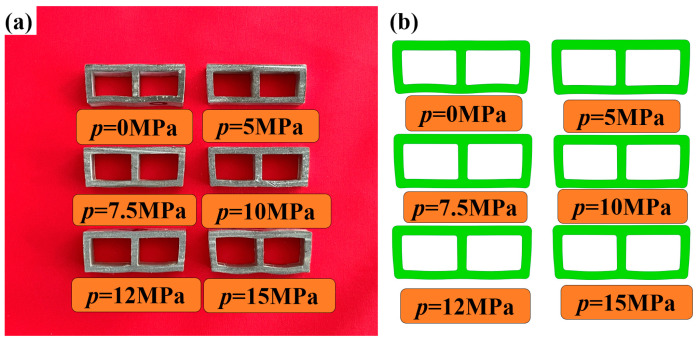
Comparison of experimental and simulated bending sections: (**a**) experimental bending section; (**b**) simulated bending section.

To verify the feasibility and accuracy of the finite element model for double-lumen tubes under liquid-filled bending, a comparative study was conducted between the finite element analysis results and experimental results. Taking the first specimen of each group as an example, Figure 16 shows the bending cross-section morphology obtained from both the experiments and finite element analysis under different internal pressures.

Table 4 presents the depression depths of all bent tubes determined by experiments and finite element analysis. The results show that the maximum relative error between the simulated and experimental values was 13%, with a minimum relative error of 2.4% and an average error of 6.02%. The present finite element model demonstrated acceptable accuracy in predicting the bending deformation of double-lumen tubes under different internal pressures of fluid filling.

### 6.2. Experimental Study of Cross-Sectional Distortion of Tubing under Different Internal Pressures

**Figure 17:**
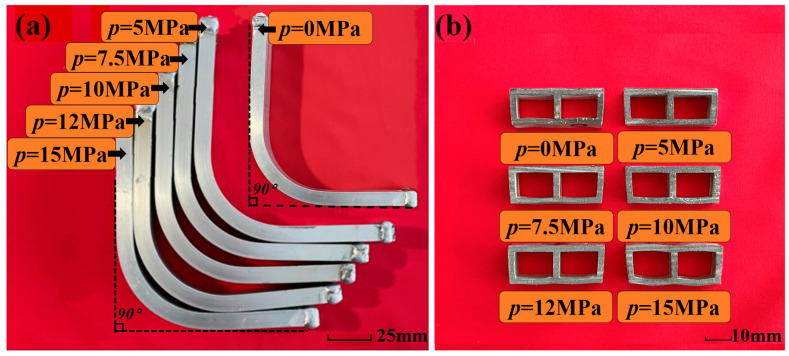
Experimental parts: (**a**) billet forming part; (**b**) typical cross-sectional section at different internal pressures.

To illustrate the influence of internal pressure, the first specimen from each group, representing different internal pressure conditions, was analyzed. Figure 17a shows the tube billet forming parts, while Figure 17b presents the cross-sectional shape of the liquid-filled bending deformation area. This cross-sectional shape was obtained by intercepting the geometric central section of the deformation zone of the double-lumen tube. The analysis reveals the impact of internal pressure on the cross-sectional shape of the deformation zone during bending.

**Figure 18:**
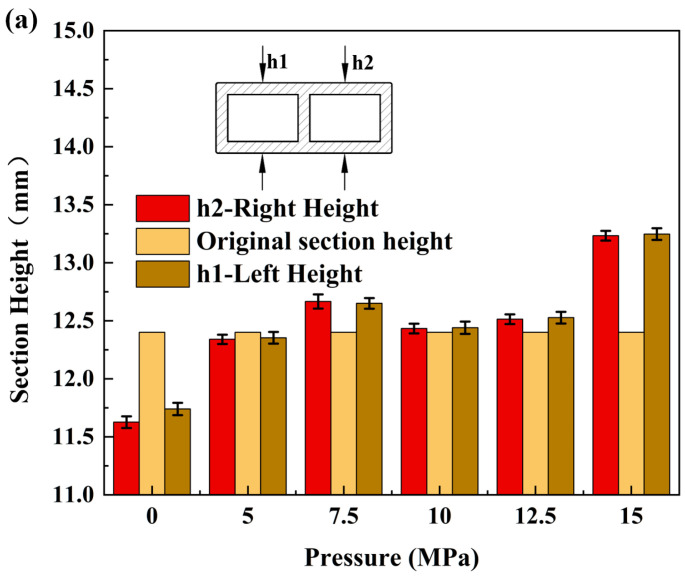

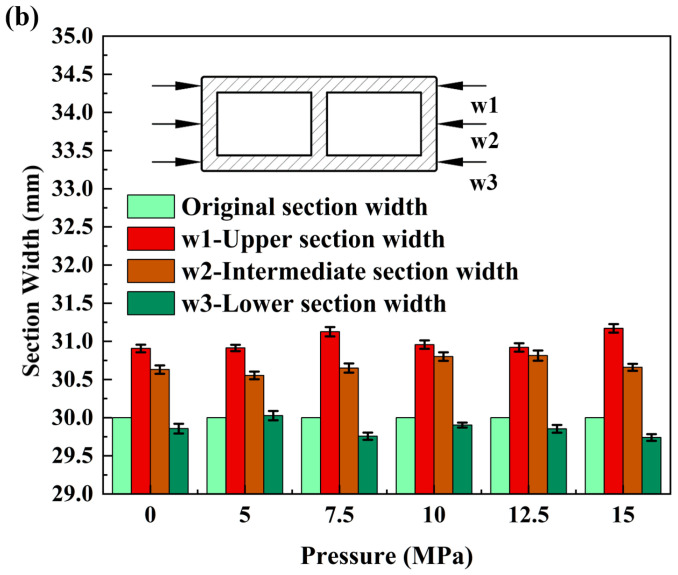
Dimensional changes of billet-formed parts: (**a**) change in height of billet-formed part section; (**b**) change in width of billet-formed part section.

The individual measurements for each group of three test pieces were analyzed and averaged to obtain a representative value for each group. The changes in the cross-sectional size of the billet due to bending deformation are illustrated in Figure 18. As the liquid filling pressure of the billet increased, the wall deformation transformed from concave to convex, resulting in significant improvement of concave defects. Furthermore, the internal width of the bending wall decreased due to extrusion, while the external width of the bending wall expanded, leading to an increase in the overall billet width.

Figure 17 shows the bending deformations conducted under various internal pressure support conditions, including without internal pressure. Upon examination of the figure, it is evident that when *p* = 0 MPa, the double-lumen tube exhibited a degree of inward depression on both the inner and outer surfaces of the bend. Notably, the outer surface displayed the most significant depression in the double-lumen tube deformation, while the inner surface showed minor instances of such defects. Additionally, the inner side of the fascia plate thickened, with a noticeable thinning change occurring on the outer side.

When *p* = 5 MPa, the inward concave defects in the double-lumen tube bending improved compared to those at *p* = 0 MPa. This suggests that the internal support pressure of the liquid-filled double-lumen tube during the bending and forming process had a suppressive effect on the concave defects on both inner and outer surfaces of the bend.

When *p* = 7.5 MPa, there was a substantial reduction in the inward concavity on the outer surface of the double-lumen tube bend compared to the stage without internal pressure, resulting in an almost horizontal outer wall in the outer zone. Observing the change in wall curvature, it was clear that the liquid’s support on the tube wall progressively increased with internal pressure, effectively curbing the formation of inward concave defects.

When the pressure *p* reached 10 MPa, the outer wall near the fascia area became completely flat, while the curvature of the outer wall near the two rounded corners remained negative. Meanwhile, the inner wall was tightly pressed against the bending mold, approximating a straight line. As the pressure p continued to increase above 12.5 MPa and reached 15 MPa, the curvature of the outer wall changed from negative to positive, and its value increased gradually. Notably, in the lower left and lower right rounded corners, a clear torsion was observed due to the gradual outward expansion of the outer wall, resulting in a twisted appearance.

**Figure 19:**
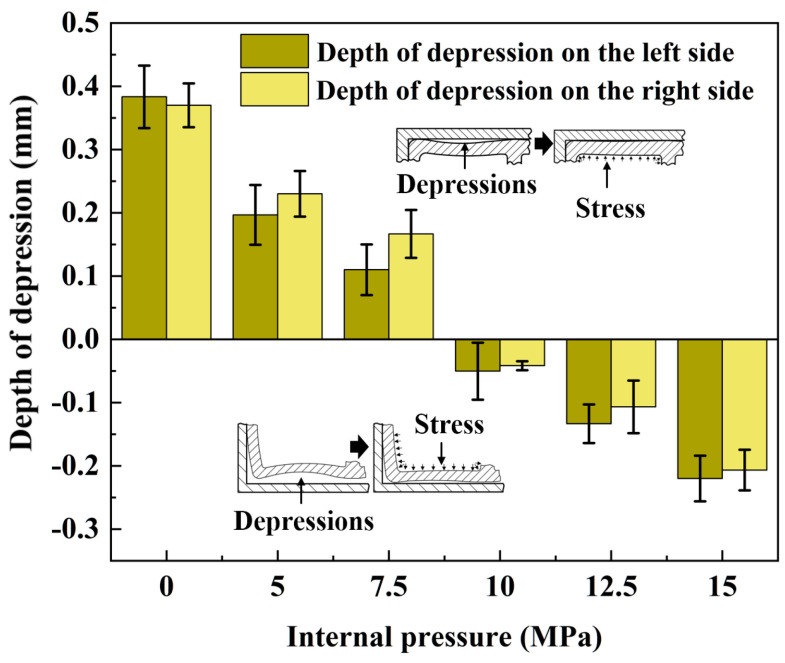
Effect of internal pressure on the depth of depression in the tube wall.

Figure 19 shows the depth of concavity of the outer tube wall under various internal pressures. To ensure measurement accuracy, the depth of concavity on both sides of the outer tube wall was measured simultaneously during the testing process. The figure reveals that as the liquid pressure within the double-lumen tube increased, the overall concavity of the outer tube wall underwent a transition from a decrease in inward concavity to an increase in outward concavity, with the concavity becoming negative.

When the internal pressure shifted from 0 MPa to 5 MPa, the depth of concavity on the left side decreased from 0.36 mm to 0.18 mm, representing a 50% reduction, which was the most significant decrease in concavity. Similarly, the depth of concavity on the right side decreased from 0.35 mm to 0.22 mm, resulting in a 37% decrease, further supporting the conclusions derived from the external contour plot in Figure 17. The most pronounced change in contour amplitude occurred when transitioning from 0 MPa to 5 MPa.

The broadside unadherence rate is a crucial metric for evaluating the degree of distortion in the cross-section of double-lumen tubes during bending and forming processes. It quantifies the extent to which the side walls deviate from the bending mold. The bending mold’s limiting effect on the tube wall near the inner part of the rounded corner can be observed during the bending of tube fittings. This results in tangential compressive stress thickening and an increase in material, which is then transferred circumferentially. As the thickness increases, the material tightly adheres to the bending mold. Conversely, the outer part of the tube wall experiences tangential tensile stress during bending, leading to tensile reduction of the material and deviation from the bending mold, causing distortion on the inner side of the tube wall and the outer side of the tube wall.

**Figure 20:**
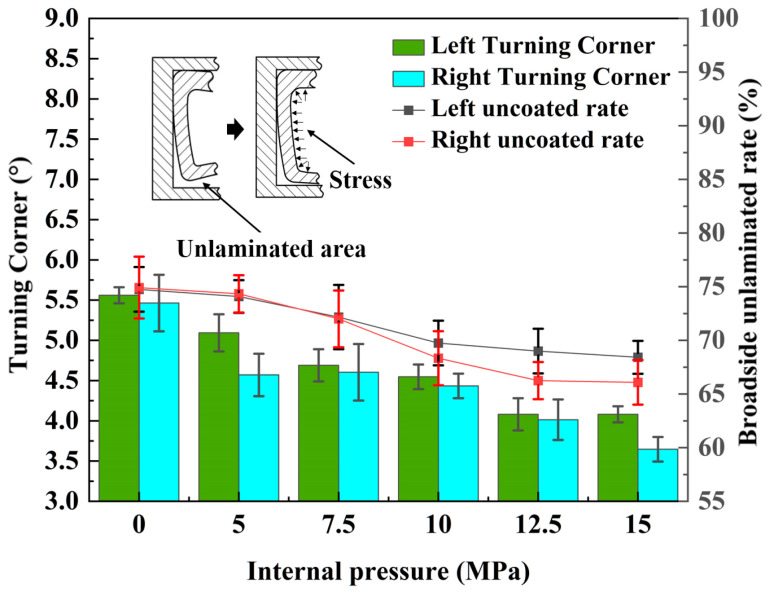
Effect of internal pressure on broadside unapplied molding rate and rounded corner turnout.

The length of the molded lateral tube wall, $h_{min}$, was measured to determine the broadside unmolded rate, calculated as $h^{\prime }=(\left(h-h_{min})/h\right)$, as illustrated in Figure 20. The angle of rotation of the unapplied mold wall after the side wall was pulled by the outer wall, denoted by *ψ*, serving as a measure for the degree of cross-sectional distortion in double-lumen tube bending and forming.

With the gradual increase in internal pressure, the unmolded rate on both left and right sides decreased. Specifically, the left side’s unmolded rate dropped from 74.08% to 66.75%, achieving an optimization rate of 7.33%, while the right side’s unmolded rate declined from 76.58% to 66.75%, attaining an optimization rate of 9.83%. Furthermore, the angles on both sides also decreased as the internal pressure rose. The left side angle diminished from 5.56° to 3.98°, yielding an optimization rate of 28.42%, and the right side angle decreased from 5.43° (unfilled liquid) to 3.68° at 15 MPa, resulting in an optimization rate of 32.23%.

**Figure 21:**
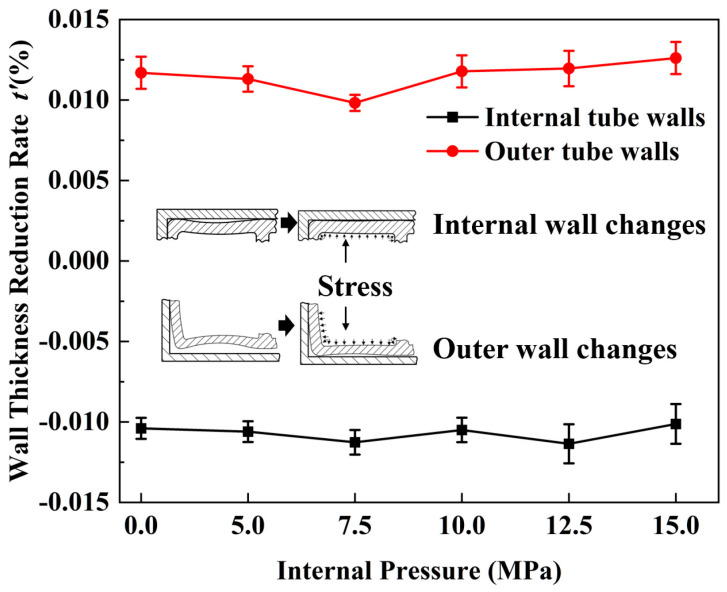
Wall thickness thinning rate of tube wall at variable internal pressures.

Figure 21 shows the wall thickness thinning rates of the inner and outer tube walls at various internal pressures. The outer wall exhibited a high wall thinning rate due to its complex deformation behavior. In contrast, the inner wall exhibited a low wall thinning rate, which tended to decrease and then increase with increasing internal pressure. The reason for this phenomenon is that as the pressure continued to rise, the inwardly recessed wall was corrected and re-adhered to the inner surface of the bending mold. At *p* = 7.5 MPa, the inner wall was largely corrected. However, as the internal pressure continued to rise, the outer wall of the formed tube, unrestricted by the mold, tended to transform the double-cavity rectangular tube into a double-cavity round tube, causing the inner wall to warp with negative curvature, subjected to circumferential tensile stresses, resulting in relative thinning of the wall thickness.

Compared to the previously studied fluid-filled bending of fittings without internal reinforcement, the fluid-filled bending of rectangular cross-section fittings with reinforcement was successfully formed. Within a suitable range of internal pressure, both the depression in the bending section and the form of the internal reinforcement remained stable. Furthermore, the variation in wall thickness of the billet after forming was reduced. These findings provide ancillary evidence that liquid-filled bending of billets from double-lumen aluminum alloy tubes can have a beneficial, assisting, or protective effect on the deformation of the billet.

### 6.3. Numerical Simulation of Wall Thickness Distribution in Bending of Tubing under Different Internal Pressures

**Figure 22:**
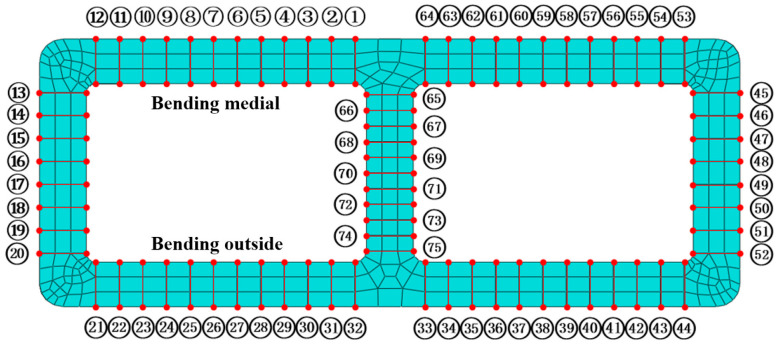
Measuring points of billet section.

When there was no liquid pressure inside the tube, the wall thickness distribution of the inner wall of the double-lumen tube was symmetric, with the wall thickening from node ① to node ⑤ and then thinning, a thickening peak appearing at node ②, and the trough of the curve emerging from node ④ to node ⑦. The other side also displayed the same distribution. The curve trend for *p* = 0 MPa was due to the absence of internal pressure support inside the tube. After bending the double-lumen tube via mold extrusion, the axial stress was compressive, causing the material at the tube ends to be extruded towards the middle and an increase in inner wall material. However, the tube cross-section length was limited by the bending mold, preventing material expansion in the length direction. Consequently, the tube wall became concave towards the lumen due to the lack of internal pressure support, resulting in the measurement of nodes ① and ⑫ displaying a slight decrease in wall thickness and the generation of a small circumferential tensile strain in the tube wall. For the same reason, the tube wall, which was concave to the lumen, bent at the center of the tube wall under plastic deformation, generating a positive bending moment, and the wall thickness decreased in comparison to other areas due to the external pressure and internal tension of the material, with the nodes shown in Figure 22.

**Figure 23:**
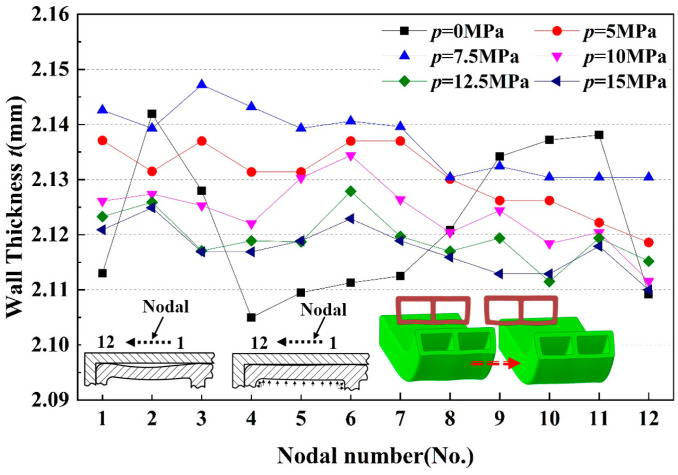
Distribution of wall thickness at each characteristic point of the inner tube wall at different internal pressures.

Figure 23 shows the wall thickness distribution of the inner tube wall. A notable variation in wall thickness distribution was observed at different internal pressures. When the two-lumen tube was subjected to internal liquid pressure (*p* > 0 MPa), the plastic deformation behavior of the inner tube wall was altered. At *p* = 5 MPa, the symmetric stress profile resulting from inward bending of the inner wall, observed in the absence of internal pressure, was significantly improved, and the wall thickness at all points was more uniform. As the internal pressure continued to increase, the average wall thickness remained stable, but the wall thickness varied under different internal pressures. From a macroscopic perspective, the order of wall thickness was $t_{7.5}^{\prime }>t_{5}^{\prime }>t_{10}^{\prime }>t_{12.5}^{\prime }>t_{15}^{\prime }$. The reason for this is that as the pressure increased, the inwardly recessed wall was corrected and re-adhered to the inner surface of the bending mold. At *p* = 7.5 MPa, the inner wall was largely corrected. However, as the internal pressure continued to rise, the outer wall of the formed tube, unrestricted by the mold, tended to transform the double-cavity rectangular tube into a double-cavity round tube. Consequently, the inner wall experienced negative curvature warping, subjected to circumferential tensile stresses, resulting in relative thinning of the wall thickness.

**Figure 24:**
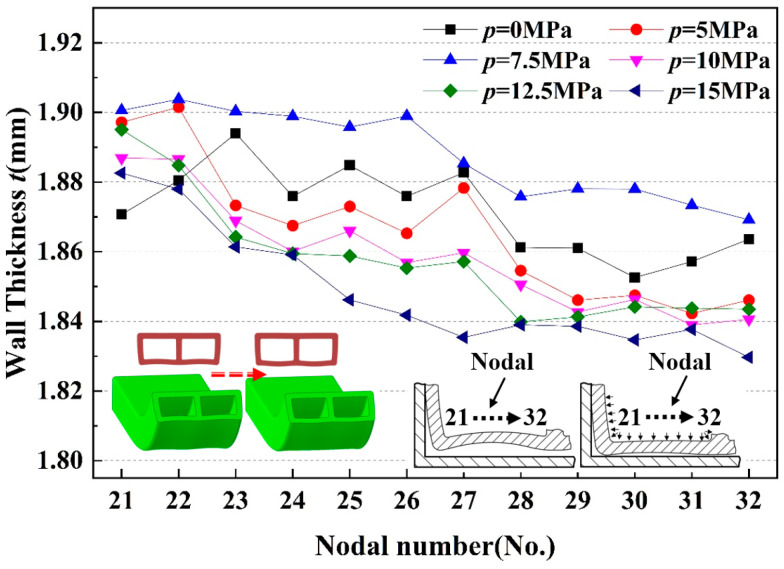
Distribution of wall thickness at each characteristic point of the outer tube wall under different internal pressures.

Figure 24 shows the wall thickness distribution of the outer tube wall. As the internal pressure increased, the wall thickness first rose and then decreased, following the order of $t_{7.5}^{\prime }>t_{5}^{\prime }>t_{10}^{\prime }>t_{12.5}^{\prime }>t_{15}^{\prime }$. This phenomenon occurred because, as the pressure continued to rise, the inwardly concave outer wall was pushed back to a flat state by the internal pressure and then began to protrude outward. When the internal pressure kept increasing, the double-lumen tube, which featured open forming characteristics due to its winding and bending process, lacked mold restrictions at the end of the outer wall formation. Consequently, the double-lumen rectangular tube transformed into a double-lumen round tube as the internal pressure rose. As a result, the outer wall experienced an increase in circumferential tensile stress due to the higher internal pressure, leading to a relative thinning of the wall thickness.

**Figure 25:**
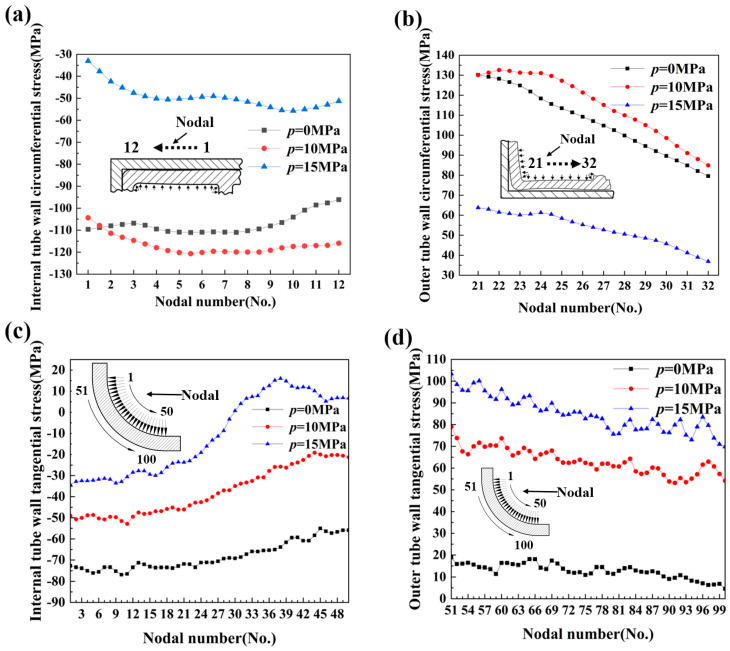
Distribution of inner and outer axial stresses under different internal pressures: (**a**) internal tube wall circumferential stress; (**b**) outer tube wall circumferential stress; (**c**) internal tube wall tangential stress; (**d**) external tube wall tangential stress.

Figure 25a,b shows the circumferential stress distribution on the inner and outer sides of a typical cross-section of a bent double-lumen tube at various internal pressures. It can be observed that the inner circumferential stresses were compressive, while the outer circumferential stresses were tensile. Furthermore, the magnitude of the circumferential stresses followed a specific pattern: the stresses at 10 MPa exceeded those at 0 MPa, which in turn exceeded those at 15 MPa, regardless of whether it was the inside or the outside. Figure 25c,d shows the internal and external tangential stresses at different internal pressures. The tangential stresses exhibited a consistent pattern, with compressive stresses on the inner side and tensile stresses on the outer side, and their values increased with increasing internal pressure, following the order: 15 MPa > 10 MPa > 0 MPa. As the internal liquid pressure rose, the positive pressure on the tube wall against the bending mold increased, leading to greater friction during the bending process. In this bending process, the billet rotated around the bending mold, experiencing sliding friction with the inner mold and rolling friction with the outer roller. The increase in inner and outer stresses caused by the increased friction was one of the reasons for the change in tangential stress under different internal pressures.

### 6.4. Simulation of Bending Behavior in Tubing with Variable Wall Thicknesses

**Figure 26:**
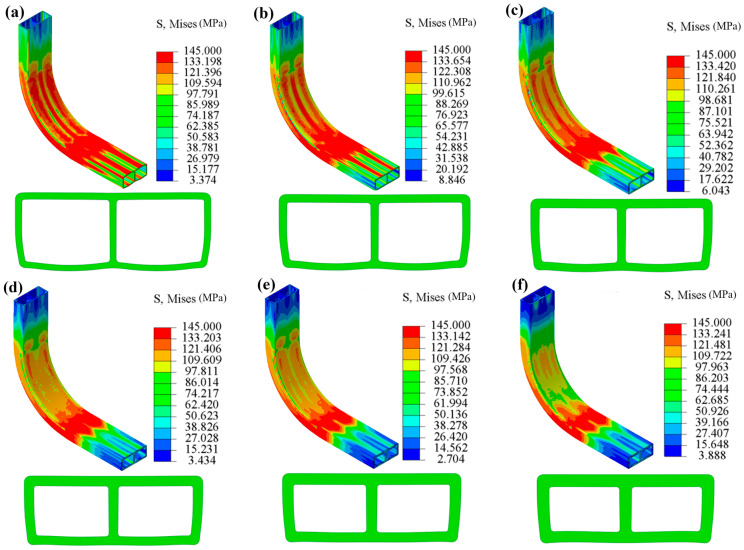
Stresses and cross-sectional shapes of bent parts with varying wall thicknesses: (**a**) *t* = 1 mm; (**b**) *t* = 1.2 mm; (**c**) *t* = 1.4 mm; (**d**) *t* = 1.6 mm; (**e**) *t* = 1.8 mm; (**f**) *t* = 2 mm.

Numerical simulations of hydraulic bending of double-lumen tubes were performed in ABAQUS finite element software under an internal pressure of 5 MPa and a bending radius of 50 mm. The simulations considered six different wall thicknesses of tube billets: 1 mm, 1.2 mm, 1.4 mm, 1.6 mm, 1.8 mm, and 2 mm. The resulting equivalent stresses and cross-sectional shapes of the intercepted bending and forming parts are presented in Figure 26.

From the figure, it can be observed that when *t* = 1 mm, the outer wall exhibited a positive curvature curve shape, expanding outward, while the inner wall also expanded outward, displaying a negative curvature shape, albeit with a significantly smaller curvature size due to the constraint of the bending mold. Furthermore, when the radius remained unchanged and the wall thickness decreased, the required internal pressure for deformation also decreased. As shown in Figure 26, as the wall thickness of the billet gradually increased, the degree of outward expansion of the tube wall decreased. Conversely, when the wall thickness increased, the internal pressure required for deformation rose. Moreover, the same internal pressure had different effects on double-lumen tubes with varying wall thicknesses. Notably, when *t* = 2 mm, the curvature of the outer wall changed from positive to negative, transitioning from outward expansion to inward depression.

**Figure 27:**
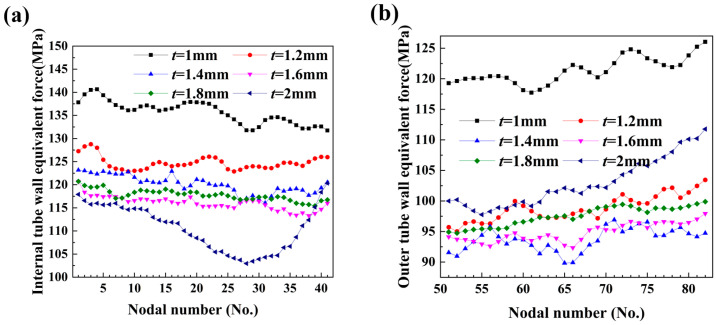
Equivalent force distribution for different wall thicknesses: (**a**) internal tube wall equivalent force; (**b**) external tube wall equivalent force.

As shown in Figure 26, the equivalent stress cloud for fittings with different wall thicknesses revealed that the stress on the inside of the bend in the central bending portion decreased gradually with increasing wall thickness. This trend is further supported by Figure 27a, which illustrates that the equivalent force on the inner side of the bent fittings also decreased with increasing wall thickness. The increase in wall thickness led to an increase in the internal pressure required for deformation, and for thicker billets, the same internal pressure resulted in a smaller magnitude of change in the billet.

The outer wall exhibited a negative curvature state, subjected to thick compressive stress from the bending mold, tangential tensile stress from the bending deformation, and tensile stress from the two lateral edges, resulting in a relatively large equivalent stress. Meanwhile, it can be observed that when *t* > 1 mm, the equivalent force of the outer wall did not deviate significantly from that of the inner side, as the curvature change of the outer wall was relatively small, leading to a small change in the equivalent force, as illustrated in Figure 27b.

### 6.5. Effect of Different Bending Radii on the Wall Thickness of Tube Fittings

**Figure 28:**
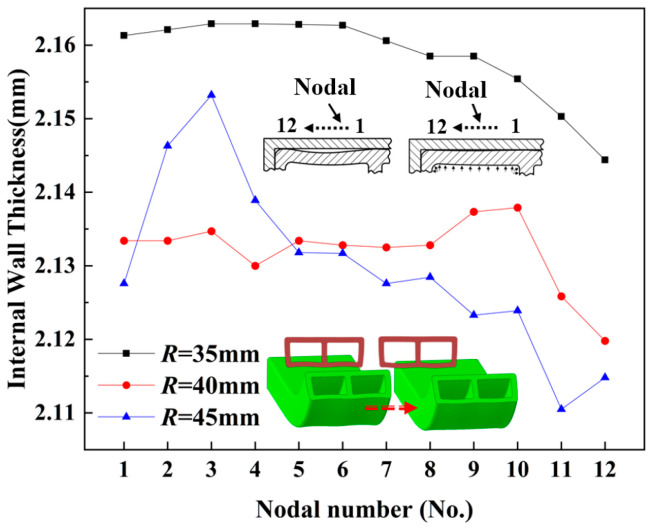
Distribution of wall thickness of inner tube wall at different bending radii.

**Figure 29:**
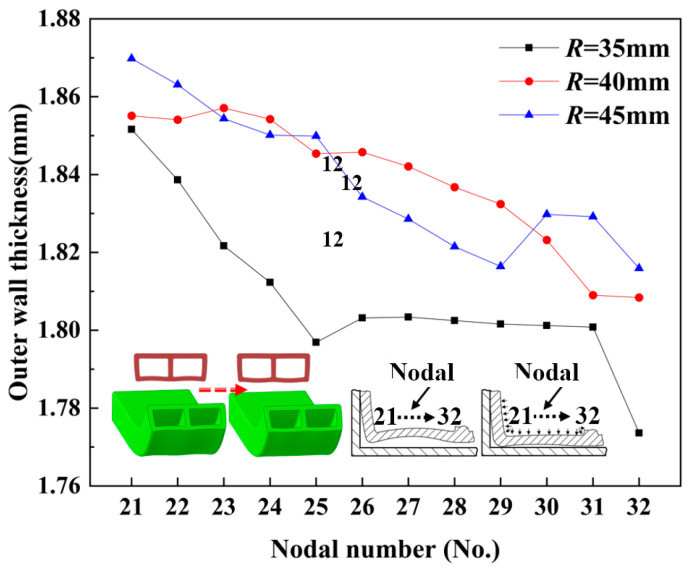
Wall thickness distribution of the outer tube wall at different bending radii.

Figure 28 and Figure 29 show the wall thickness distribution of the inner tube wall at different bending radii within the bending deformation zone of the bent part, under the same internal pressure (*p* = 5 MPa) and the same wall thickness (*t* = 2 mm). As observed in Figure 28, the wall thickness when *R* = 35 mm was significantly higher than that when R = 40 mm and *R* = 45 mm. The highest wall thickness of the former was 1.4% greater than the median wall thickness of the latter two, resulting in a distinct distribution curve compared to the overlapping wall thickness distribution curves of the latter two.

This phenomenon occurred because the lower bending radius resulted in a larger curvature of the bend, leading to a more severe degree of material buildup on the inside of the bend. Consequently, the wall thickness on the inside of the bend was considerably higher than that of the other two bending radii in the bent parts.

As shown in Figure 29, the outer wall thickness of the *R* = 35 mm bend was smaller than that of the R = 40 mm and *R* = 45 mm bends. Specifically, the median wall thickness of the *R* = 35 mm bend was 2.4% smaller than that of the *R* = 40 mm bend and 1.6% smaller than that of the *R* = 45 mm bend. Furthermore, the wall thickness distribution curves of the *R* = 40 mm and *R* = 45 mm bends overlapped. This phenomenon can be attributed to the greater bending curvature of the outer wall of the *R* = 35 mm bending fitting compared to the other two bending radii. As a result, during the bending process, the outer side of the *R* = 35 mm bend experienced higher tensile stress, leading to more severe material loss and a higher wall thickness thinning rate than the other two.

## 7. Conclusions

The liquid-filled bending forming method proposed in this study effectively mitigated cross-sectional distortion defects in the low-pressure liquid-filled bending process of aluminum alloy double-lumen tubes. Through a combination of experimental investigation and numerical simulation, the deformation and stress characteristics of the liquid-filled bending process were thoroughly analyzed, yielding the following key conclusions:

(1) Through a combination of liquid-filled bending experiments and numerical simulation methods, this study reveals the effects of internal pressure on the wall thickness distribution, cross-sectional distortion parameters, and stress distribution of formed tube fittings. The results show that increasing the internal pressure improved wall thickness uniformity, with a 2.51% improvement in outer wall straightening from 0 to 7.5 MPa. The wall thickness distribution followed an arch-shaped pattern, peaking at 7.5 MPa. As internal pressure increased, the inner concave of the tube wall was initially corrected to a straight state, then became convex, while the outer wall rotated around the rounded area to become straight, resulting in a rounded cross-sectional shape. Furthermore, the rates of un-posted molds on both sides decreased gradually with increasing internal pressure, with optimization rates of 7.33% and 9.83%, respectively. Additionally, the corner angles on both sides decreased with increasing internal pressure, with optimization rates of 28.42% and 32.23%, respectively. These findings demonstrate that internal pressure can significantly improve molding quality during bending and forming.

(2) The relationship between the tube billet wall thickness and the deformation of the bending tube was established. The size of the billet wall thickness was positively correlated with the depth of the concave wall, with t = 1.8 mm serving as the demarcation point between concave and convex shapes. The outer equivalent force in the deformation zone was negatively correlated with the wall thickness, meaning that a smaller wall thickness resulted in a higher equivalent force on the cross-section. When the wall thickness was reduced to 1 mm, the equivalent force increased significantly by 7.8% compared to 1.2 mm, and the equivalent force at t = 1 mm was 28.5% higher than that at t = 2 mm. Improving the distribution of the equivalent force and enhancing the molding quality was achieved by increasing the wall thickness.

(3) The wall thickness distribution law under different bending radii revealed that for bending radii R < 40 mm, the degree of inner thickening and outer thinning was significantly increased. The wall thickness at R = 35 mm was 1.4% higher than that at R = 45 mm, and the difference between the median wall thicknesses at R = 45 mm and R = 40 mm was no more than 0.1%. As the bending radius narrowed, the reduction of inner and outer materials became more pronounced, subsequently increasing the difficulty of molding.

## Figures and Tables

**Table 1:** 6063-T5 aluminum alloy tubing chemical composition.

Elements	Fe	Cu	Mn	Mg	Cr	Zn	Ti	Al
Quantity contained (wt.%)	0.35	0.10	0.10	0.51	0.10	0.10	0.10	Balance

**Table 2:** Mechanical properties of 6063-T5 aluminum alloy tubing.

Material	$\mathrm{Yield}\;\mathrm{Strength}\;\sigma_{s}$ (MPa)	$\mathrm{Tensile}\;\mathrm{Strength}\;\sigma_{b}$ (MPa)	Strain Hardening Index n	Strength Coefficient K (MPa)	Elongation δ (%)
6063-T5	130	175	0.15	259.2	8

**Table 3:** Double-lumen tube filling and bending research program.

Groups	Fixed Parameter	Variation Parameters
Group 1	Bend radius R = 50 mm Wall thickness t = 2 mm	Internal pressure (MPa) *p* = 0, 5, 7.5, 10, 12.5, 15
Group 2	Internal pressure *p* = 5 MPa Wall thickness t = 2 mm	Bend radius (mm) R = 35, 40, 45
Group 3	Internal pressure *p* = 5 MPa Bend radius R = 50 mm	Wall thickness (mm) t = 1, 1.2, 1.4, 1.6, 1.8, 2

**Table 4:** Comparison of experimental and finite element analysis.

Group	Pressure (MPa)	Depth of Depression (mm)		Relative Error	
		Exp.	Num.	Max	Min
1	*p* = 0 MPa	0.36	0.39	8.3%	2.6%
		0.42			
		0.38			
2	*p* = 5 MPa	0.18	0.195	8.3%	2.5%
		0.20			
		0.185			
3	*p* = 7.5 MPa	0.11	0.113	5.8%	2.7%
		0.118			
		0.12			
4	*p* = 10 MPa	−0.045	−0.04	12.5%	2.4%
		−0.041			
		−0.043			
5	*p* = 12.5 MPa	−0.14	−0.13	13%	7.1%
		−0.12			
		−0.115			
6	*p* = 15 MPa	−0.25	−0.24	4%	3.2%
		−0.26			
		−0.248			

Relative error = (Num. − Exp.)/Exp. × 100%.

## Data Availability

The original contributions presented in the study are included in the article, further inquiries can be directed to the corresponding author.
